# Dermal Perfusion of Common Donor Sites Free Flaps in Chronic Smokers and Nonsmokers

**Published:** 2011-12-19

**Authors:** Afshin Rahmanian-Schwarz, Viktor Molnar, Rennekampff H.-O., Phillipp Gonser, Lina Willkomm, Amro Amr, Manuel Held, Hans-Eberhard Schaller, Hirt Bernhard

**Affiliations:** ^a^Department of Plastic, Reconstructive, Hand and Burn Surgery, BG-Trauma Center, Eberhard Karls University Tuebingen, Germany; ^b^Department of Plastic, Hand, Aesthetic and Burn Surgery, Stiftungsklinikum Mittelrhein, Koblenz, Germany; ^c^Department of Plastic Surgery, Hand and Burns Surgery, University Aachen, Germany

## Abstract

**Objective:** The smoking behavior of the patient influences the indication of plastic surgeon in his reconstruction procedure on the assumption that smoking may increase the complication risks. In the present study, we evaluate the particular aspect of topographic differences in dermal perfusion in chronic smokers and nonsmokers. **Methods:** The perfusion parameter of 8 common donor sites for free flap transplantation were investigated in 152 smoking and nonsmoking subjects (*n* = 152; women: *n* = 78, 51%; men: *n* = 74, 49%; smokers: *n* = 38, 25%; nonsmokers: *n* = 114, 75%) using the O2C device (LAE Medizintechnik Giessen GmbH, Gießen, Germany). Oxygen saturation (%), relative hemoglobin concentration (AU [arbitrary unit]), Velocity (AU) and Flow (AU) were monitored noninvasively and compared. **Results:** All monitored regions did not show any significant differences in parameters oxygen saturation (smokers = 40%, nonsmokers = 44.5%), relative hemoglobin concentration (smokers = 60 AU, nonsmokers = 60 AU), flow (smokers = 19.5 AU, nonsmokers = 16.5 AU) and velocity (smokers = 10 AU, nonsmokers = 10 AU) between chronic smoking and nonsmoking subjects (*P* < .05). Also, a distinction between smokers and nonsmokers as a function of gender (women: *n* = 78, 51%; men: *n* = 74, 49%) showed no significant differences in all 4 parameters. **Conclusions:** Varied statements regarding surgical complications in chronic and acute smokers were described in the literature. This raises the question of how far restricting the indication of reconstruction procedure for smoking patients due to higher complications is justified. In our study, there is no significant drop of dermal perfusion parameters after chronic tobacco consumption. Nonetheless, the unfavorable effects of smoking in general to human body and health remain undoubted.

Transplantation of free flaps has become a common and essential tool for the closure of large defects in reconstructive surgery. Flap failure, tissue necrosis, hematoma formation, impaired wound healing, and prolonged recovery time of patients are the most frequently reported complications after free flap transfers.^1^^,^^2^ According to literature, these complications occur significantly more often in acute smoking patients than in nonsmoking patients.^3^^-^6 Searching for causes acute smoking is not only known to lead to local vasoconstriction by increasing skin sympathetic activity and to promote endothelial dysfunction but also to decrease tissue blood flow, oxygen tension, and aerobe metabolism.^7^ As it is reported that the success rate of free flap transfers depends on the donor site,^8^ many surgeons think of tobacco consumption as a generally contraindication for free flap transplantation.^9^ Considering the adverse aspects of smoking on the outcome of surgery in addition to broad clinical experience, some surgeons even claim the right to deny elective surgery to heavy smokers.^10^^,^^11^

However, patients often need a plastic surgery reconstruction, which are chronic smokers. Furthermore, varied statements regarding surgical complications in chronic smokers were described in the literature. To scrutinize these views and the unfavorable effects of chronic tobacco consumption on the cutaneous microcirculation, this study aims at investigating topographic differences in dermal perfusion in 152 female and male chronic smokers and nonsmokers using the O2C device (LEA Medizintechnik Giessen GmbH, Gießen, Germany).

## METHODS

The measurements were carried out in 2009 using the O2C device. On the basis of a multichannel system, the instrument uses the reflection of emitted white light to detect oxygen saturation (sO_2_ in %) and relative hemoglobin concentration (rHb in AU [arbitrary unit]), and the reflection of emitted laser light to calculate flow (F in AU) and velocity (V in AU) of corpuscular structures within the vessels such as erythrocytes.^12^ The method performs a noninvasive measurement of all four parameters at 2 mm depth.

The study population consisted of 152 test persons (*n* = 152; women: *n* = 78, 51%; men: *n* = 74, 49%) at an average age of 32.6 (18-75) years and included 38 subjects (*n* = 38, 25%) who declared to regularly smoke tobacco (up to 20 or more cigarettes a day). Exclusion criteria were vascular and dermatological diseases, hypertonia, diabetes mellitus, and anticoagulative medication. Patients were advised to quit smoking 24 hours before our measurements. Table 1 shows the demographic data and inclusion criteria of our test persons.

Eight regions of the body which are commonly used as donor sites in free flap transplantation and the forehead which is known as one of the best perfused regions of the body^13^ have been chosen as regions of interest (Table 2). Two standardized measurements of each region in a distance of 7 days were taken on the laying test persons after resting for at least 5 minutes to calculate average values.

In addition to the aforementioned parameters, data regarding age, gender, weight, arterial pressure, and heart frequency of all subjects were collected.^14^

All data were processed using JMP software (Version 5.1) for analyses of regression and variance. *P* < .05 was considered to indicate statistically significant differences.

## RESULTS

**Figure 1:**
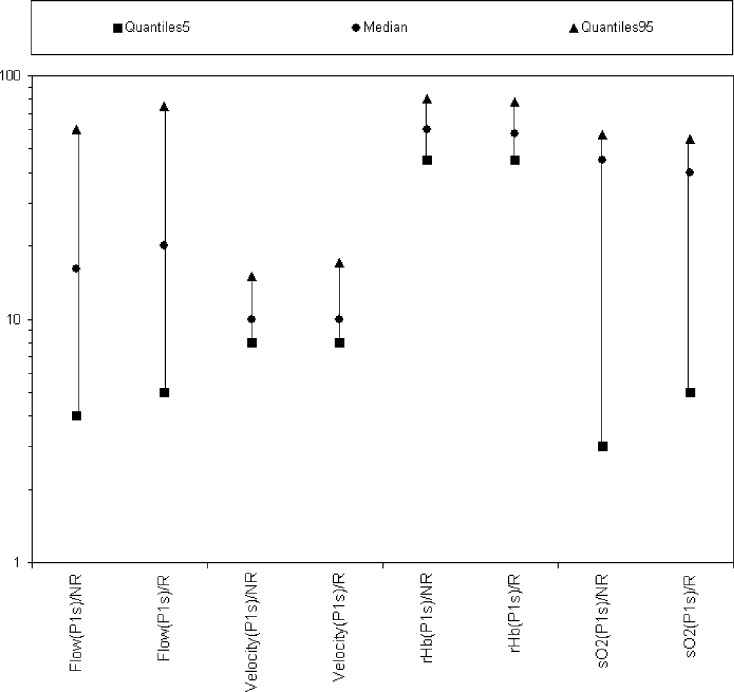
Parameters of smoking (n = 38) and nonsmoking (n = 114) subjects in relation to smoking status. Smokers and nonsmokers were compared in the individual parameters. The reference values of the parameters lie between the 5% and 95% quantile.

**Figure 2:**
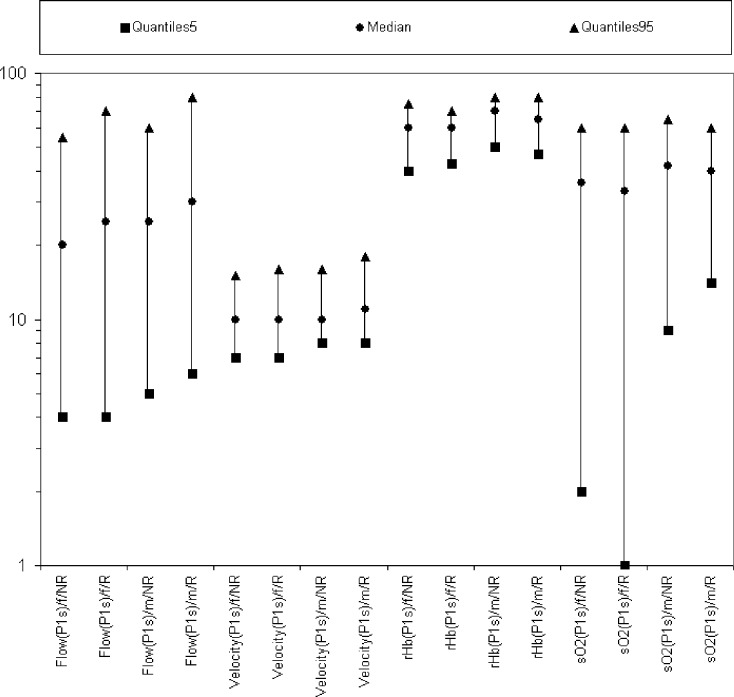
Representation of the parameters of all smokers (n = 38) and nonsmokers (n = 114) as a function of gender (women: n = 78, men: n = 74). The reference values of the parameters lie between the 5% and 95% quantile.

The main finding of this study is that comparing male and female chronic smokers (*n* = 38, 25%) to nonsmokers (*n* = 114, 75%) there were no significant differences in the 4 parameters oxygen saturation (sO2%), relative hemoglobin concentration (rHb [AU]), flow (F [AU]), and velocity (V [AU]) measured at 2-mm depth in the aforementioned 9 regions. (Fig 1) Also, a distinction between smokers and nonsmokers as a function of gender (women: *n* = 78, 51%; men: *n* = 74, 49%) showed no significant differences in all 4 parameters. (Fig 2).

Even comparing the measurements of each parameter of every single region of smokers and nonsmokers the findings were similar. A significant difference in none of the parameters could be found. These results could not be associated in any way to age, weight, arterial pressure, or heart frequency of the test persons.

## DISCUSSION

The adverse effects of acute tobacco consumption on cutaneous microcirculation have been investigated in previous studies^15^ and showed increased blood pressure,^16^ decreased tissue blood flow, and oxygen tension^7^ especially in young smokers.^17^ With longer duration of tobacco consumption, the negative effects and impairment of acute smoking on dermal perfusion grows weaker.^17^ Chronic smokers' microcirculation seems to become inured to smoke^18^ and a generalized microvascular vasomotor dysfunction^19^ with disturbed peripheral microcirculation^20^ is assumed.

On the assumption that smoking increases the complication risks, some surgeons claim the right to deny elective surgery to heavy smokers. Unfortunately, the majority of patients needing plastic surgery reconstructions are chronic smokers. However, considering the different statements in the literature on the effects of chronic smoking, the question arises how far a restraint regarding indication of reconstruction procedure for these patients is justified.

In this context, we monitored the effects of chronic tobacco consumption of perfusion parameter of common donor sites for flap transplantation in 152 smoking and nonsmoking subjects. Excluding patients with additional disease such as hypertonia and vascular disorder from our study groups, we focused our evaluation of the isolated effect of smoking. The measurement method with the O2C device has already been validated by Walter et al^21^ in an experimental work and showed a very good correlation coefficient. O2C is a suitable and easy method for monitoring tissue oxygenation and blood flow noninvasive and simultaneously.^22^ After defining individual basic values for all parameters for every subject using the O2C device intraindividual changes and disturbances could be performed very easily and precisely.

The highest flow and concentration of hemoglobin was found in the region of the forehead and the dorsum of the hand. This goes along with the findings of Stucker et al,^13^ who observed a remarkably higher bloodflow in the face compared to all other regions of the body.^13^ In concordance with the study of Wolff et al, a poor oxygenation was found in the paraumbilical region in both smokers and nonsmokers.^23^

In 1996, Kroll et al^8^ claimed that the success rate of free flap transfers depends on the donor site. Following Kroll's assumption, we decided to evaluate common donor sites for free flap transplantation regarding their potential. Furthermore, we assessed the dermal perfusion of the donor site as an important indicator for flap perfusion and flap survival.

Thinking of dermal perfusion as a function of gender, Stucker et al^13^ and Maurel et al^24^ reported differences in dermal perfusion in women and men, whereas Park et al^25^ report no differences. In our study, gender had no influence on dermal perfusion in women and men no matter if the subject smoked or not.

Analyzing the association between chronic smoking and complications following skin surgery, Dixon et al^26^ even claimed smokers and nonsmokers to suffer complications similarly.

Considering our results and the previously mentioned literature, the negative effects of acute tobacco consumption appear more likely to be responsible for surgery's outcome, which goes along with the findings of Bianchi et al^1^ and van Adrichem et al.^27^ That is why a preoperative smoking cessation of at least 4 weeks is recommended in elective surgery to significantly reduce the risk of postoperative complications and increase the statistical probability of flap survival.^28^^,^^29^

If a preoperative smoking cessation is not possible, patients should be advised to immediately quit smoking,^30^ as smoking in the perioperative period should absolutely be avoided.^31^ The patients have to be offered professional counseling to reach this goal.^10^

Also confirming our results, in basal condition, Rossi et al^32^ did not observe any significant difference in spectral intensity of skin flowmotion between smokers and nonsmokers. However, they observed an absent postischemic increase of blood flowmotion in the *cutaneous* microcirculation of healthy chronic cigarette smokers together with a relatively preserved skin postischemic hyperemia Regarding ischemia-reperfusion injuries as well as known complication of flap transplantations, this particular aspect should be investigated in further studies concerning a possible influence.

## CONCLUSION

Summing up, the results of this study suggest the conclusion that chronic tobacco consumption does not lead to decreased dermal perfusion in any of the investigated 9 regions of the body of women or men in comparison with nonsmokers. On the basis of our assessed parameters, chronic tobacco consumption has not been verified to be a contraindication for flap transfers generally. In addition, we believe that our results provide a valuable baseline data, which are indispensable for further specific investigation regarding the smoking effect of different flap types. Nevertheless, the harmful effects of chronic smoking for the human body and health should not be underestimated.

## Figures and Tables

**Table 1:** Demographic data and characteristics of all smoking and nonsmoking subjects*

	Total	Smokers	Nonsmokers
Quantity, n (%)	152 (100)	38 (25)	114 (75)
Female, n (%)	78 (51)	16	62
Male, n (%)	74 (49)	22	52
Age, median (range), y	32.6 (18-75)		
Heart frequency, median (range), bpm	72 (54-96)		
Middle arterial pressure, median (range), mm Hg	100 (70-120)		
Female			
Weight, median (range), kg	66.5 (49-100)		
BMI, median (range)	23.8 (19.1-35)		
Male			
Weight, median (range), kg	82 (60-106)		
BMI, median (range)	25.6 (19.6-35.1)		
Cigarettes per day (average)		20	
Pack years (average)		4.5	
Time period between last cigarette and surgery, h		24	

*BMI indicates body mass index.

**Table 2:** Parameters depending on body region and gender (medians: female/male) for smokers (s) and nonsmokers (ns)*

	sO_2_ (s)	sO_2_ (ns)	rHb (s)	rHb (ns)	F (s)	F (ns)	V (s)	V (ns)
Forehead	58/50	50/55	68/74	66/76	50/64	37/47	14/14	13/14
Upper arm	40/45	43/51	58/62	60/66	16/11	14/13	8/8	8/10
p. Forearm	39/41	40/49	56/63	58/66	23/28	13/17	10/10	8/8
d. Forearm	45/49	48/52	61/70	60/72	17/22	12/18	8/10	8/10
Back of the hand	44/48	48/53	67/76	67/74	42/37	32/35	13/12	11/12
Supraumbilical	13/31	13/16	53/55	56/59	11/25	16/16	8/8	10/8
Thigh	37/42	36/47	50/55	57/62	14/16	15/17	10/10	10/10
Shank	41/38	27/41	52/55	54/63	12/17	8/12	8/8	8/10
Scapula	38/44	39/42	56/63	59/63	16/13	14/18	8/8	9/10

*F indicates flow; rHb, relative hemoglobin concentration; sO_2_, oxygen saturation; V, velocity.
